# Handwerkschirurgie im frühen 17. Jahrhundert. Das Praxisjournal eines Münsteraner Barbiers

**DOI:** 10.1007/s00048-023-00372-z

**Published:** 2024-01-04

**Authors:** Michael Stolberg

**Affiliations:** Universität Würzburg, Würzburg, Deutschland

**Keywords:** Barbiere, Wundärzte, Chirurgie, Medizinische Praxis, Frühneuzeitliche Medizin, Praxisjournal, Münster, Barber-surgeons, Surgery, Medical practice, Early modern medicine, Practice journal, Münster

## Abstract

Der Beitrag stellt das Praxisjournal eines Münsteraner Barbiers vor, in dem dieser rund 950 Fälle verzeichnete, die zwischen 1602 und 1614 in seine Behandlung kamen. Gestützt auf diese Quelle, untersucht er die Klientel, die Behandlungsanlässe und die Honorarforderungen eines handwerklich gebildeten Wundarztes im frühen 17. Jahrhundert.

## Abstract (en)

This paper presents and analyzes the practice journal of a barber-surgeon in the town of Münster, in Northern Germany, in which he recorded about 950 cases he treated between 1602 and 1614. Based on this source, it examines the clientele and the fees of a German barber-surgeon in the early seventeenth century, and looks at the injuries and complaints for which patients sought his treatment.

## Einleitung

Die handwerklich gebildeten Barbiere, damals oft auch „Barbierer“, „Balbierer“ oder „Scherer“ genannt, waren zusammen mit den Badern (Flamm 1996; Widmann & Mörgeli 1998) eine wichtige Säule der frühneuzeitlichen Gesundheitsversorgung. In den größeren Städten überstieg ihre Zahl die der studierten Ärzte nicht selten um ein Vielfaches. Vielerorts schlossen sie sich zu Zünften, Gilden oder Bruderschaften zusammen und waren als Vertreter eines angesehenen Handwerksberufs fest im städtischen Leben verankert (Kinzelbach 2014; Kinzelbach 2016). Sogenannte „Amtsrollen“ oder Zunftordnungen regelten die inneren Verhältnisse, die Ausbildung der Lehrlinge und Gesellen, die Prüfungen sowie die Rechte und Pflichten der Mitglieder.^1^

Wir verfügen mittlerweile über eine ganze Reihe von älteren und neueren Arbeiten zu den Badern und Barbieren in einzelnen Städten des Reichsgebiets, die eingehend die jeweiligen Bader- und Barbiersordnungen untersuchen und ergänzend Ratsprotokolle und andere städtische Verwaltungsquellen zu Rate ziehen.^2^ Sie bieten wertvolle Aufschlüsse über die zünftische Organisation der Bader und Barbiere, ihre Auseinandersetzungen mit Vertretern anderer Heilberufe und ihre Interaktionen mit den örtlichen Autoritäten. Nur ganz vereinzelt und anekdotisch eröffnen solche primär normativen und administrativen Quellen und die überlieferten Zunftakten jedoch konkretere Aufschlüsse über die alltägliche Praxis dieser handwerklich gebildeten Wundärzte. Zentrale Fragen bleiben so in der Forschung weitgehend ungeklärt: Wie sah ihr Arbeitsalltag aus? Wieviel verlangten sie für ihre Dienste? Wer waren ihre Patientinnen und Patienten? Welche Krankheiten und Verletzungen behandelten sie? Inwieweit nahmen sie auch größere chirurgische Eingriffe vor wie die Operation von Eingeweidebrüchen und Blasensteinen? In welchem Ausmaß behandelten sie, gegebenenfalls auch gegen obrigkeitliche Verbote, innere Krankheiten und verabreichten Arzneien, die die Kranken einnehmen mussten? Inwieweit gaben sie sich mit Haareschneiden und Rasieren ab? Zu Recht beklagte Annemarie Kinzelbach noch vor nicht allzu langer Zeit die bislang „äußerst lückenhafte Kenntnis konkreter chirurgisch-medizinischer Praktiken, die im vormodernen Alltag eine Rolle spielten“ (Kinzelbach 2017: 250).

Die bislang unbekannte Quelle, die in diesem Beitrag vorgestellt und untersucht werden soll, ist vor diesem Hintergrund von herausragendem Wert.^3^ Es ist das Praxisjournal und Rechnungsbuch eines Münsteraner Barbiers für die Jahre 1602 bis 1614. Praxisjournale haben in der jüngeren Forschung als Quelle für die Untersuchung der alltäglichen heilkundlichen Praxis vergangener Zeiten einige Aufmerksamkeit gefunden (Dinges et al. 2016). Selbst aus ärztlicher Feder sind sie jedoch aus dem 16. und 17. Jahrhundert nur sporadisch überliefert (König 1961; Assion & Telle 1972; Schlegelmilch 2018; Stolberg 2019), und die vorliegende Quelle ist das erste und einzige bislang im Reichsgebiet identifizierte Praxisjournal eines handwerklich gebildeten Wundarztes aus der Zeit vor 1700 überhaupt.^4^ Wie wir sehen werden, lässt auch diese Quelle manche Fragen offen. Mit ihren Einträgen zu fast tausend Behandlungsfällen gibt sie jedoch erstmals einen umfassenden Einblick in die Praxis eines städtischen Barbiers jener Zeit, über die Patientinnen und Patienten, die zu ihm kamen, die Verletzungen und sonstigen Leiden, die sie zu ihm führten, und das Entgelt, das er für seine Dienste verlangte.

## Die Münsteraner Barbierszunft

Ehe wir uns diesen Aufzeichnungen im Detail zuwenden, gilt es zunächst kurz den lokalen Kontext zu skizzieren. Die Einwohnerzahl Münsters belief sich 1591 nach den Berechnungen, die Franz Lethmate anhand von Steuerlisten und Feuerstättenverzeichnissen angestellt hat, auf 10.617; darunter waren 3933 Kinder (Lethmate 1912: 34). Die Anzahl der Barbiere, die damals in Münster praktizierten, ist nicht genau bekannt, aber in den Ratsprotokollen und in anderen städtischen Aktenbeständen der Zeit lassen sich zwischen 1602 und 1614 insgesamt zwölf Barbiere identifizieren: Hermann Hölscher senior und junior, Johann Hölscher, Henrich Graes, Gerrit und Hermann von Vorden (auch: Vörden), Kaspar Lethmate, Bernd Westeken, Hans Deist, Melchior Rökelose und zwei Barbiere namens Bernd Lange, von denen einer 1609 starb und der andere noch Jahre später dokumentiert ist – vermutlich also Vater und Sohn. Die Aufzählung könnte vollständig sein: 1685 führte das Münsteraner Kopfschatzungsregister elf Barbierschirurgen auf (Mai 1995: 67). In Köln kamen Ende des 16. Jahrhunderts auf einen Barbier rund 1160 Einwohner (Jütte 1989: 190). In Rostock mit einer mit der von Münster vergleichbaren Bevölkerungszahl waren im 16. und 17. Jahrhundert in der Regel rund zehn Barbiersmeister tätig (von Brunn 1921: 11).

Die Barbiere waren auch in Münster auf jeden Fall die mit Abstand größte Gruppe unter den approbierten Heilkundigen. Bader mit eigenen Badstuben sind für das frühe 17. Jahrhundert nur zwei aktenkundig (Gördes 1917: 52) und es sind auch nur zwei promovierte Ärzte bekannt, die im Zeitraum zwischen 1602 und 1614 in Münster praktizierten. Zunächst war Georg Moll(en) sogar der einzige studierte Arzt in Münster, bis 1603 Bernhard Rottendorff nach Münster zurückkehrte (Gördes 1917: 14 f.).

Die Münsteraner Barbiere waren in der „Broderschafft der Barberer [sic!] und sembttlicher Verwanten der Chyurgischer [sic!] Kunst“ organisiert, deren auf der Grundlage der Kölner Barbiersordnung von 1397 verfasste „Amtsrolle“ der Rat im November 1564 genehmigt hatte.^5^ Der Rat sicherte sich jedoch wichtige Kontrollbefugnisse. Er war es, der alljährlich aus den Reihen der Bruderschaft die beiden Zunftvorsteher oder „Verweser“ ernannte, und die Bestimmungen der Amtsrolle durften nur mit seiner Zustimmung verändert werden. Die Lehrzeit wurde in der Amtsrolle grundsätzlich auf drei Jahre festgelegt, konnte aber verkürzt werden, wenn der Betreffende bereits das nötige Wissen und Geschick erworben hatte. Nach Abschluss der Lehrzeit musste er drei Jahre in Münster oder andernorts bei einem oder mehreren Meistern arbeiten. Die üblichen Wanderjahre als Geselle waren also nicht verpflichtend vorgesehen. Die Meisterprüfung wurde durch die beiden Verweser und zwei weitere gewählte Meister abgehalten.

Grundsätzlich sollte die Behandlung von Kranken und Verwundeten in Münster nur den einheimischen Meistern zustehen. Vermutlich auf Betreiben des Rats stellte die Amtsrolle jedoch sicher, dass die Hilfesuchenden in der Wahl ihrer Behandler weiterhin große Freiheiten genossen. Den fahrenden Heilern, den sogenannten „Landttreckers“ oder „Landtloperß“, war es zwar verboten, in der Stadt ihre Salben, Balsame, Öle und sonstigen Mittel anzubieten und zu verkaufen. Der Rat konnte aber geschickten und erfahrenen Meistern gestatten, ihre Dienste auch außerhalb der drei „freien“, unregulierten Märkte anzubieten, die zweimal jährlich zu den sogenannten „Synoden“ sowie an Peter und Pauli abgehalten wurden. Patienten, die an Wassersucht, Steinen, einem Bruch, einer Verbrennung oder anderen Gebrechen litten, war es zudem ausdrücklich gestattet, auch einen fremden, auswärtigen Meister zu konsultieren. Wenn es darum ging, gebrochene Gliedmaßen einzurichten und andere, nicht näher benannte Leiden zu behandeln, durften sie sich sogar einem Scharfrichter anvertrauen – Scharfrichter waren damals häufig auch medizinisch-chirurgisch tätig (Harrington 2014: 271–324; Schefknecht 2021). Als die Barbiere 1609 über den Schinder Wessel klagten, der sich zum Nachteil der Barbiere „unzimlich der Beinbrüche und anderer Schäden curirung“ unterfange, wurde dieser dagegen ernsthaft ermahnt, solches in Zukunft zu unterlassen.^6^

## Das Journal und sein Verfasser

Der Katalog des Nordrhein-Westfälischen Landesarchivs in Münster führt im Bestand des Vereins für Geschichte und Altertumskunde Westfalens das „Journal eines münsterschen Arztes“ auf. Es handelt sich um ein kleines Bändchen im Format von circa 18,5 × 14,5 cm mit knapp 130 Blättern, zusammengebunden mit einem Blatt aus einer liturgischen Handschrift. Es konnte ursprünglich mit zwei Lederbändchen verschlossen werden. Der Verfasser war offenkundig kein studierter Arzt, sondern ein Barbier oder Wundarzt. Dieser verzeichnete in weitestgehend chronologischer Reihenfolge die Patientinnen und Patienten, die er zwischen 1602 und 1614 behandelte, dazu einige Männer, die er regelmäßig rasierte.^7^ Für die vorliegende Untersuchung habe ich sämtliche Einträge jeweils mit dem Datum, dem Namen und dem Geschlecht der Behandelten und, soweit angegeben, auch den Wohnort, den Beruf oder sozialen Status, das bezahlte oder ggf. noch ausstehende Honorar sowie die Diagnose und gegebenenfalls die Verletzungsursache tabellarisch erfasst und statistisch ausgewertet.

**Figure 1:**
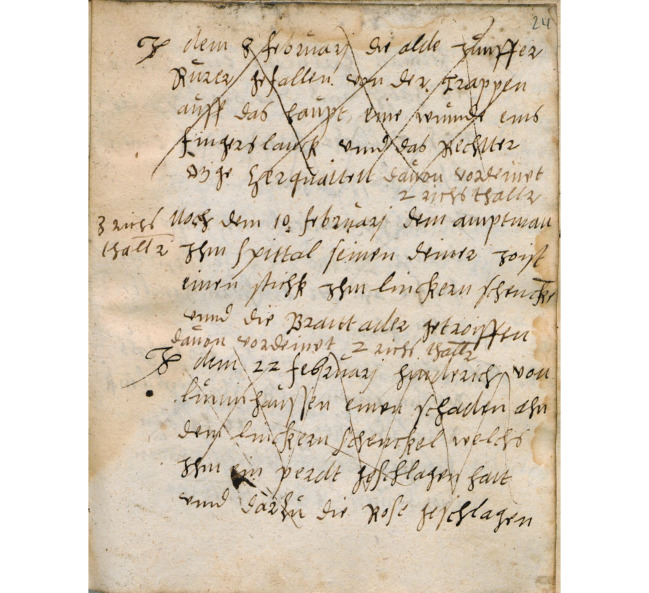

Die Eintragungen sind in einer flüssigen, insgesamt sauberen Handschrift gehalten (siehe Abb. 1). Schreibweise und Begriffswahl spiegeln den lokalen Sprachgebrauch, beispielsweise mit „boven“ für „über“ und „zerquatz(t)“ für „gequetscht“ oder „verletzt“. Allem Anschein nach verfügte der Schreiber nur über eine begrenzte formale Schulbildung. Nicht nur bei Eigennamen und diagnostischen Begriffen, sondern auch bei Wörtern des alltäglichen Sprachgebrauchs variiert häufig die Schreibweise. Lateinische Fachbegriffe finden sich nur ausnahmsweise und in einer teilweise sehr eigenwilligen Schreibweise, die erkennen lässt, dass der Schreiber sie aus dem mündlichen Austausch kannte – vermutlich mit einem Lehrmeister oder Arbeitskollegen – und nicht aus der Lektüre einschlägiger Schriften. So steht „superstoriumm“ (fol. 38r) für „suppositorium“ (Zäpfchen) und „hermoroides“ (fol. 58v) für Hämorrhoiden. Selbst bekannte Fachbegriffe wie „podagra“ oder „paralyse“, die damals auch unter gebildeten Laien durchaus gebräuchlich waren, fehlen; der Schreiber gab „Cipperlein“ und „Rorung“ („Rührung“, für „Schlaganfall“) den Vorzug.

Das Journal trägt keinen Besitzvermerk und es ist mir bisher nicht gelungen, den Verfasser sicher zu identifizieren. Er erwähnte seine Frau (fol. 121v) und schließlich seine „selige Hausfraw“ (fol. 124r). 1609 verzeichnete er zudem, zusätzlich zu seinem eigenen Honorar, die Zahlung von einem Taler an seinen Sohn für die Behandlung einer verwundeten Magd (fol. 71r), was darauf schließen lässt, dass er damals bereits einen erwachsenen Sohn hatte, der selbst als Barbier tätig war oder zumindest gerade das Barbiershandwerk erlernte. All diese Angaben würden zu Hermann Hölscher dem Jüngeren passen, der ab 1602 auch über Jahre als einer der beiden Zunftverweser fungierte. Selbst Sohn eines gleichnamigen Barbiers hatte er seinerseits einen Sohn, Johann Hölscher, der ebenfalls als Barbier dokumentiert ist. 1618, nach dem Ende der Aufzeichnungen, lebte er bei diesem Sohn, hatte also keinen eigenen Hausstand mehr und war somit vermutlich ein Witwer in fortgeschrittenem Alter.^8^ Andere der oben genannten Münsteraner Barbiere lassen sich jedoch als mögliche Verfasser nicht ausschließen.^9^

Eine zentrale Funktion des Journals war zweifellos die Buchführung. Mit seiner Hilfe wollte der Barbier offenbar den Überblick über die geleisteten und vor allem die noch ausstehenden Zahlungen behalten, die er noch einfordern konnte, von den Patienten und Patientinnen selbst oder gegebenenfalls von den Erben. Zwar finden sich nur in rund einem Drittel der Einträge konkrete Angaben zum Honorar. Fast alle Einträge wurden jedoch, so wie wir das aus anderen zeitgenössischen Rechnungsbüchern kennen, mit kreuzweise geführten Linien so durchgestrichen, dass sie weiterhin lesbar blieben. Hier war die Honorarfrage allem Anschein nach erledigt, und wenn der Barbier keine konkreten Angaben machte, hatte er das Honorar vermutlich auf der Stelle erhalten oder vielleicht auch in manchen Fällen darauf verzichtet. Die zahlreichen Erläuterungen zu den mit konkreten Zahlen versehenen Eintragungen sind nämlich regelmäßig in der Vergangenheit gehalten und benennen manchmal konkret den Zeitraum, über den der Verfasser den jeweiligen Patientinnen und Patienten „gefolgt“ war. Offensichtlich machte er diese Einträge also erst, wenn die Behandlung abgeschlossen war. Zu diesem Zeitpunkt, so ist zu vermuten, stellte er, wenn dies noch erforderlich war, seine Honorarforderung, je nach der Zahl der Besuche beim Patienten und den Kosten der abgegebenen Arzneien. An einer Stelle erwähnt das Journal in diesem Sinne ausdrücklich die „Rechnung“, die er einem Junker übergeben hatte (fol. 120r). Dagegen finden sich keinerlei Hinweise auf Behandlungsverträge oder feste Honorarvereinbarungen zu Beginn der Behandlung, wie sie beispielsweise aus Zürich überliefert sind (Wehrli 1927: 68).

Die teilweise recht präzisen diagnostischen Angaben weisen zugleich über eine finanzbuchhalterische Funktion im engeren Sinne hinaus. Das Journal verzeichnet beispielsweise häufig die Art und Weise, wie es zu einer bestimmten Verletzung gekommen war und ob der rechte oder der linke Arm beziehungsweise das rechte oder das linke Bein etc. betroffen war, wie lange eine Schnittwunde war und dergleichen mehr. Dagegen ist die Art der Behandlung nur teilweise und der Behandlungserfolg lediglich in Einzelfällen dokumentiert. Das lässt vermuten, dass der Barbier das Journal auch führte, um Rechenschaft über die von ihm behandelten Fälle ablegen zu können. Möglicherweise geschah das auch vor dem Hintergrund einer Meldepflicht für schwere Verletzungen, die der Münsteraner Rat Anfang 1602 beschloss,^10^ genau zu dem Zeitpunkt also, zu dem die Aufzeichnungen einsetzen.

## Die Klientel

Das Journal verzeichnet für die rund zwölfeinhalb Jahre vom Februar 1602 bis zum September 1614 insgesamt 948 Behandlungsfälle, also durchschnittlich 76 Fälle im Jahr. Die Zahl der behandelten Patientinnen und Patienten war mit insgesamt rund 790 oder durchschnittlich 63 pro Jahr geringer, denn ungefähr 160 Einträge – die Zahl lässt sich nicht genau bestimmen^11^ – beziehen sich auf Ratsuchende, für die das Journal mehrfach eine Behandlung verzeichnet, manchmal innerhalb kurzer Zeit, manchmal in jahrelangem Abstand. Die nachfolgenden Zahlenangaben beziehen sich grundsätzlich auf die 948 Behandlungsfälle. Vermutlich – wir werden darauf zurückkommen – handelte es sich hierbei um jene Fälle, die der Barbier selbständig behandelte und in der Regel auch mehrmals sah, denn Einträge zu Patientinnen und Patienten, die er nur zur Ader ließ oder denen er einen Zahn zog, fehlen völlig.

In 937 der 948 Fälle lässt sich das Geschlecht aus dem Vornamen oder aus anderen Angaben erschließen. Demnach entfielen 69 % (647) auf Buben und Männer und 31 % (290) auf Mädchen und Frauen. Männliche Patienten überwogen also deutlich. Ähnliches kennen wir, wenn auch nicht immer ganz so ausgeprägt, aus zeitgenössischen ärztlichen Praxisjournalen und Rezepttagebüchern. So betrug der Anteil der männlichen Patienten in den Praxen von Hiob Finzel, Georg Palm und Petrus Kirstenius respektive 56,6 %, 63,4 % und 70,7 % (Ofenhitzer 2015; Stolberg 2019, fig. 3). Das Alter ist nicht angegeben, doch Bezeichnungen wie „Sonneken“, „Dochterken“ oder „Kindeken“ lassen immerhin erkennen, dass mindestens 50 der Behandlungen auf Säuglinge und kleinere Kinder entfielen, davon 14 auf Mädchen, 25 auf Buben und 11 auf Kinder, deren Geschlecht nicht eindeutig zu erkennen ist.^12^

In den Einträgen zu 404 Behandlungsfällen finden sich Hinweise auf den beruflichen und/oder sozialen Status der Hilfesuchenden beziehungsweise auf den des Vaters, des Ehemanns oder eines Geschwisters, oder dieser Status lässt sich anderen Einträgen zu den Betreffenden oder ihren Angehörigen entnehmen. Das Spektrum ist breit. 45 Einträge lassen sich einem Junker und 18 einem (Dom‑)Herren und deren jeweiligen Familien zuordnen. 1 Patient war Komtur. 71 Einträge verweisen auf wichtige Ämter, wie Drost (4), Gaugraf (11), Domdekan (11), Sekretär des Domkapitels (9), Schulze (7), Procurator (9), Richter (7) Amtmann (11) und Gerichtsschreiber (2). Mindestens 126 von 948 Behandlungsfällen oder gut 13 % entfielen somit auf Mitglieder aus Oberschichtsfamilien. In 37 Einträgen (4 % der Gesamtzahl der Behandlungsfälle) werden Patienten durch einen akademischen Titel gekennzeichnet und lassen sich somit im weiteren Sinne dem Bildungsbürgertum zurechnen, nämlich Lizentiaten (24), Magister (2), Doctores (10) und ein Professor. Die Zahl der Geistlichen war dagegen mit 4 Patres, 4 Pastoren und 3 Pröbsten auffällig klein. Vermutlich waren allerdings auch manche der mit ihren akademischen Titeln angeführten Patienten – insbesondere die Lizentiaten – Geistliche. In 9 weiteren Fällen verweisen Einträge als „Herr“ und in 2 weiteren als „Bürger“ bzw. „ehrwürdige“ Frau mittelbar auf Angehörige der höheren Schichten.

Bei Adligen und Akademikern war es damals üblich, jeweils den Titel zu nennen, so dass die Zahlen für diese Gruppe vermutlich ziemlich vollständig waren. Sie hätten somit immerhin rund ein Sechstel der Klientel gestellt. Für die übrige Bevölkerung sind die Angaben weniger eindeutig. So fällt auf, dass Händler nicht ausdrücklich als solche erwähnt sind dabei war Münster war eine blühende Handelsstadt. In 70 Fällen lässt eine konkrete Berufsbezeichnung wie Hufschmied, Goldschmied, Kleinschnittler (Tischler), Schreiner, Schlachter, Mühlenmeister, Bäcker, Buchbinder, Sattelmacher, Schmied, Schneider oder Schuhmacher, ein „M.“ für „Meister“ oder beides erkennen, dass die Hilfesuchenden aus Handwerkerkreisen stammten. Dazu kommen 7 „Jungen“, eine geläufige Bezeichnung für „Lehrjungen“, und 9 Baumeister oder „Bauschulzen“ lassen sich vermutlich zumindest im weiteren Sinne ebenfalls den Handwerkern zurechnen.

In 112 Fällen verweisen die Einträge auf einfaches Dienstpersonal, nämlich 56-mal auf Mägde, 28-mal auf Diener und 24-mal auf Knechte; dazu kommen 1 Jäger und 1 Schweinehirt. Oft dürfte hier der – meist ausdrücklich genannte – Dienstherr die Kosten für die Behandlung seiner Bediensteten übernommen haben. Von einer Magd mit einem „Schaden“ am Knie heißt es allerdings ausdrücklich, dass ein halber Taler von ihrem Lohn einbehalten wurde (fol. 47v). Dazu kommen niedrigere Amtsbedienstete, nämlich 1 Stabträger (Gerichtsgehilfe), 2 Boten, 1 Nachtwächter und 2 „Kustoden“ (in Nienberge und Bösensell). Nur 2 Soldaten kamen in Behandlung. Drei weitere Einträge beziehen sich auf die vermutlich kostenlose Behandlung von Frauen im Armenhaus.

Die Zahlen lassen nur vorsichtige Schlüsse zu, nicht zuletzt wegen fehlender Daten für die Gesamtbevölkerung. Die mittleren und oberen Schichten stellten vermutlich einen überproportional großen Anteil, zumal wenn man auch ihr Dienstpersonal hinzurechnet. Für die Annahme, dass die Barbiere die Heilkundigen des „gemeinen Manns“ waren, während die höheren Schichten die Dienste eines promovierten Arztes bevorzugten, bietet das Journal keinerlei Belege. In Fragen der Wundarznei zählte im deutschen Reichsgebiet in den Augen der Ratsuchenden offenbar nicht die theoretische Ausbildung, sondern die praktische Erfahrung und Expertise.

Die überwiegende Mehrzahl der Patientinnen und Patienten wohnte, soweit erkennbar, in Münster. Nur rund 100 Einträge^13^ verweisen ausdrücklich auf einen Wohnort außerhalb Münsters. 24 dieser Patientinnen und Patienten lebten in Nienberge, 14 in Roxel, und 2 in Albachten – Orte, die alle jeweils ca. 7 km von Münster lagen –, 4 kamen aus Wolbeck, rund 10 km von Münster, und 9 aus dem rund 14 km entfernten Bösensell. Nur ausnahmsweise wurde der Barbier aus dem weiteren Umland konsultiert, etwa aus Freckenhorst, Dülmen und Sassenberg, rund 25 km bzw. gut 30 km von Münster entfernt. Vermutlich suchten die auswärtigen Patientinnen und Patienten den Barbier zumeist in Münster auf, aber in einzelnen Fällen vermerkte er ausdrücklich, dass er ihnen auf ihren Wohnort „folgte“, wie der Frau eines Richters Teltge in Wolbeck und dem an Kopf und Hüfte verletzten Junker Johann Gras, den er sogar zweimal im rund 40 km entfernten Geisterholz besuchte.

## Behandlungsanlässe

Fast alle Einträge im Journal – 943 von 948 – benennen den jeweiligen Behandlungsanlass. Das Journal eröffnet damit einmalig differenzierte Einblicke in das Spektrum der Leiden und Verletzungen, mit denen ein Barbier im frühen 17. Jahrhundert in seiner alltäglichen Praxis konfrontiert war. Mittelbar zeigt es damit zugleich, bei welchen Leiden und Verletzungen der Barbier nicht die erste Anlaufstelle war.

Um mit dem letztgenannten Punkt zu beginnen: An keiner Stelle erwähnt das Journal die operative Behandlung von Eingeweidebrüchen (Hernien), grauem Star oder Blasensteinen, also jener drei Leiden, die damals des Öfteren Gegenstand größerer operativer Interventionen waren. Manche fahrenden Operateure waren auf solche Eingriffe spezialisiert, und einzelne Städte stellten eigens Wundärzte ein, die diese Eingriffe beherrschten. In Augsburg betrieben die Fugger sogar ein eigenes „Schneidhaus“, in dem Stein- und Bruchleidende operiert wurden (Dieminger 1999: 131–139; Kinzelbach 2021; Ruisinger 2021). Das Journal verzeichnet dagegen nur drei Steinleidende. Zweien gab der Barbier Medikamente. Dem dritten, so erläuterte er, habe er „miet Wasserganck“ gedient und ihn „zwischen nach[t] vnnd tag 4 mall gefalfet [?]“. Die Bedeutung von „gefalfet“ ließ sich bislang nicht klären, aber vermutlich führte er einen Katheter ein, was das sehr beachtliche Honorar von 10 Reichstalern erklären könnte.

Eine damals verbreitete Krankheit, die im Journal ebenfalls unerwähnt bleibt, war die „Franzosenkrankheit“. Ihre Behandlung mit Guajakholz oder Quecksilberschmieren oder -räucherungen galt, wohl auch wegen der ausgeprägten äußerlichen Veränderungen, verbreitet als eine Aufgabe der Wundärzte. So bestätigte ein Zeugnis der Tübinger Medizinischen Fakultät dem Wundarzt Georg Engel 1608, dass er so wie andere Meister des „Schererhandwercks“ neben dem Aderlass und der Behandlung von Wunden, Knochenbrüchen und ausgerenkten Gliedern auch die „HoltzChur“ erlernt habe.^14^ Das Journal erwähnt lediglich sieben Fälle mit einem „Schaden“ am „Gemächt“.^15^ Zwei weitere Patienten litten an einem „bosen Schwantz“. Einer der Patienten mit einem „Schaden“ am Gemächt war ein kleines Kind, ein anderer entwickelte eine Gangrän. Insofern ist unklar, inwieweit wir es selbst in diesen wenigen Fällen wirklich mit der Franzosenkrankheit zu tun haben.

Eine weitere „Fehlstelle“ im Journal sind frauenheilkundliche Fälle. Nur die Frau eines gewissen Cristian Monell ist mit einem einen halben Finger langen „Schaden ihn ihrer Frawlicheit“ verzeichnet (fol. 108r). Dass der Barbier die Länge des „Schadens“ in Fingerlängen präzisierte, so wie er das auch bei äußeren Verletzungen tat, lässt vermuten, dass er den „Schaden“ persönlich in Augenschein nahm. Im Einzelfall, so deutet sich damit an, waren die Frauen also bereit, sich vor dem männlichen Chirurgen zu entblößen. Eine zweite Patientin, Frau Oisen, litt an einer „Geschwolst ahm Liebe [sic!]“, also vermutlich ebenfalls im Unterleib (fol. 124r). Der Fall ist bezüglich des Schamgefühls weiblicher Patienten insofern bemerkenswert, als hier – es ist der einzige Eintrag dieser Art im gesamten Journal – „morgens vnnd abens“ nicht der Barbier selbst, sondern seine Frau der Kranken „folgte“. Bei Krankheiten der damals offenbar deutlich weniger stark schambesetzten weiblichen Brüste wurde dagegen er selbst immer wieder aktiv. Manchmal verzeichnete der Barbier hier nur die Diagnose, etwa eine „Rose“ der Brust. In etlichen Fällen erläuterte er jedoch, er habe die Brust „auffgedaen“ oder eine „schwärende“ oder „böse“ Brust behandelt, war also offensichtlich an der entblößten Brust zugange.^16^

Die fast vollständige Abwesenheit von Einträgen zu Aderlass und Zahnziehen lässt sich dagegen vermutlich aus der Funktion des Journals erklären. Der Aderlass galt im 17. Jahrhundert als eine Hauptaufgabe der Barbiere oder Wundärzte und war ihnen mancherorts, wie zum Beispiel in Regensburg (Plank 1999: 92), sogar ausdrücklich vorbehalten. In Münster führte der Chirurg J. zum Tempel bei zehn der dreizehn Fratres, deren Behandlung er 1692 in Rechnung stellte, einen Aderlass auf und einen weiteren hatte er geschröpft.^17^ Offenbar überließ man diese Tätigkeit also in Münster nicht oder jedenfalls nicht ausschließlich den Badern. Wahrscheinlich hat der Verfasser des Journals somit Aderlässe und das Reißen von Zähnen, die beide keine weitergehende Behandlung erforderten (und wohl sofort bezahlt wurden), nur nicht eigens verzeichnet. Wenn diese Annahme stimmt, wäre die durchschnittliche Zahl der Patientinnen und Patienten, an denen der Barbier tätig wurde, entsprechend noch deutlich größer gewesen als die der verzeichneten, selbstständig behandelten Krankheits- und Verletzungsfälle.

Wenden wir uns nun den Gründen zu, die die Hilfesuchenden tatsächlich dazu bewegten, sich in die Behandlung des Barbiers zu begeben. Die vieldiskutierte Problematik einer retrospektiven Diagnostik mit ihren häufig hochspekulativen oder sogar irreführenden Schlüssen (vgl. Leven 1998; Stolberg 2012) stellt sich für die Chirurgie nicht in der gleichen Schärfe. Die diagnostischen Begriffe, derer sich die damalige Chirurgie bediente, waren häufig deskriptiver Natur. Sie beschrieben äußerlich sichtbare Veränderungen. Diagnosen wie die einer ausgerenkten Schulter oder eines Geschwürs am Unterschenkel sind aus heutiger Sicht sehr viel besser nachvollziehbar als die eines doppelten Dreitagefiebers oder eines kalten, verschleimten Magens. Hier lassen sich in manchen Fällen zumindest plausible Vermutungen formulieren. Bei nicht verletzungsbedingten Leiden wäre eine systematische Übersetzung der im Journal angegebenen Diagnosen in die moderne medizinische Terminologie allerdings nicht nur mit großen Unsicherheiten behaftet. Sie würde auch den Blick auf die spezifischen Konnotationen der verwendeten Begriffe versperren, auf die von jenen der modernen Medizin grundlegend verschiedenen Annahmen über die Entstehung äußerlich sichtbarer Veränderungen auf, in und unter der Haut. Solche Veränderungen wurden damals nämlich vor allem auf das Bemühen des Körpers zurückgeführt, unreine, krankhafte Materie, die sich im Körperinneren gebildet oder angesammelt hatte, nach außen zu entleeren.

Die häufigste Einzeldiagnose war mit 252 Einträgen die eines „Schadens“. Das war in der damaligen Heilkunde ein gängiger und zugleich schillernder Begriff. Er bezeichnete Geschwüre (in diesem Sinne ist in zeitgenössischen Quellen auch von „alten Schäden“ die Rede), aber auch Verletzungen. In beiden Fällen, das war die für die Praxis entscheidende Gemeinsamkeit, war in der Regel die Hautoberfläche eröffnet – also „wund“ – und die Behandlung zielte darauf, die Läsion wieder zu verschließen. In 18 Fällen von „Schäden“ macht das Journal präzisierende Anmerkungen, die auf eine Wunde oder Quetschung oder auch auf einen Sturz oder einen Tierbiss als Ursache verweisen und damit klar erkennen lassen, dass es sich um eine Verletzung handelte. Da der Barbier andererseits bei Verletzungen oft ausdrücklich die Art und nicht selten auch den Hergang der Verletzung schilderte, dürfte es sich bei vielen, wenn nicht sogar den meisten der im Journal verzeichneten Fälle von „Schäden“, bei denen solche Angaben fehlen, aber nicht um Wunden, sondern um Geschwüre oder andere krankhafte Hautläsionen gehandelt haben. So gut wie sicher gilt das für ein rundes Dutzend von „Schäden“ in Mund und Hals sowie für einzelne Fälle, in denen die „Schäden“ auf Erfrierungen oder Verätzungen mit Säure (Scheidewasser) zurückgingen. 3 Hilfesuchende, die ein „Geschwer“ am Leib oder Bein hatten, und 8 mit einem „bösen“ Finger, einem „bösen Schwantz“, einem „bösen“ Nabel, einem „bösen Kinn“ oder einer „bösen“ Backe litten vermutlich ebenfalls an offenen Hautläsionen, also an Schäden im skizzierten Sinne. Im weiteren Sinne lassen sich auch blutenden Hämorrhoiden von 2 Männern zu den Schäden zählen; bei einem von beiden hatten diese dem Journal zufolge ein „Loich“ gebrochen. Wenn nähere Angaben zu Art oder Ursache des „Schadens“ fehlen, ist eine eindeutige Bestimmung freilich im Einzelfall unmöglich. Das gilt erst recht für die 13 Fälle, in denen der Barbier lediglich die Lokalisation des Leidens, nicht aber dessen Natur notierte.

Eine verwandte Diagnose war die „Rose“. Gemeint war offenbar nicht nur der Rotlauf (Wundrose), sondern auch allgemein eine lokale Rötung oder Entzündung. Die „Rose“ wird zuweilen als Begleiterscheinung oder Folge von „Schäden“ erwähnt und war in 18 weiteren Fällen die ausschließliche Diagnose.

Die zweite große Gruppe von Leiden, die sich an der Körperoberfläche manifestierten, bildeten die Geschwülste und Schwellungen. In 47 Fällen verzeichnet das Journal ein „apostema“. Darunter verstand man damals eine örtliche Ansammlung von Krankheitsmaterie unter oder in der Haut, aus der oft unreine, krankhafte Flüssigkeit ablief, wenn sie aufbrach oder mit dem Messer eröffnet wurde. Vermutlich würde man heute in vielen dieser Fälle einen Abszess oder ein Furunkel diagnostizieren.

In ebenfalls 47 Einträgen verzeichnete das Journal die Behandlung einer „Geschwulst“. Die Grenze zu den Apostemata war allem Anschein nach fließend. Wie die Apostemata verorteten sich die „Geschwülste“ in erster Linie am Arm oder unter der Achsel, im Nacken oder am Bein. In beiden Fällen vermerkte der Barbier zuweilen ausdrücklich, dass er die Geschwulst „öffnete“ und anschließend dafür sorgte, dass die Wunde wieder verheilte.

Zu den Schwellungen und Geschwülsten im weiteren Sinne wird man – im deutlichen Gegensatz zum heutigen Begriffsverständnis – auch die meisten der 21 Fälle von „Katarrh“ („catarra“) zählen müssen. Im Einklang mit dem Sprachgebrauch der studierten Ärzte (Stolberg 2021: 130–136) verwandte der Barbier den Begriff nämlich offenbar gleichbedeutend mit „Fluss“ für eine lokale, eine Schwellung verursachende Ansammlung von flüssiger Krankheitsmaterie. So diagnostizierte er bei einem Patienten mit geschwollenem Zahnfleisch eine „catarra“ (fol. 54v). Bei einem anderen erklärte er eine „catarra“ in den Ohren zur Ursache von Ohrensausen (fol. 57r). Eine „catarra“ konnte dem Journal zufolge aber auch in die Brust fallen, oder in den Bauch, in die Schulter oder die Beine. In den gleichen Kontext gehören damit auch die 3 Fälle von „Zipperlein“ an den Füßen. Die Krankheit war typischerweise durch harte Ablagerungen charakterisiert, die schon damals so genannten „tophi“. Die „catarra“ zur Wange und zum Ohr und das „cipperlein“ eines Amtmanns im Spital konnten insofern als Ausdruck der gleichen Erkrankung gelten (fol. 9r). Auch die 2 Fälle von Lähmung – einmal an der Zunge und einmal am Schenkel – sind vermutlich hier einzuordnen; im Falle der Zungenlähmung erwähnt der Barbier sogar ausdrücklich eine „catarra“.

Auf den Zufluss von Krankheitsmaterie führte die damalige Medizin in aller Regel auch Geschwüre und Schwellungen im Mund- und Rachenraum zurück. Der Barbier behandelte derlei Fälle in auffällig großer Zahl. Allein 21 Fälle entfielen auf ein „Mandelgeschwür“ und 14, mit leichten Variationen in der Schreibweise, auf eine „Angina s(i)quinantia“. „Squinantia“ war ein Fachbegriff für „Bräune“ oder „Halsentzündung“. Eine Liste mit deutschen Übersetzungen chirurgischer Fachausdrücke, die in einem handschriftlichen medizinischen Kompendium aus dem 16. Jahrhundert überliefert ist, übersetzt „squinantia“ in diesem Sinne mit „kelen geschwer“.^18^ Allem Anschein nach bezogen sich zudem zumindest die allermeisten der 41 Fälle von „bösem Hals“ auf Leiden im Mund- und Rachenraum; bei 6 der Einträge zu Mandelgeschwüren heißt es sogar ausdrücklich „böser Hals mit Mandelschwer“. In 2 weiteren Fällen litten die Patienten an einem „Halßschwer“ beziehungsweise an einem „inwendigen“ Schaden am Hals (fol. 109v; fol. 76r). Möglicherweise bezogen sich zudem manche der 25 Einträge zu „Schäden“, „Apostemata“ oder „Geschwülsten“ „am Hals“ auf den Rachenraum. Hier wird man allerdings in erster Linie äußerliche Veränderungen vermuten dürfen, zumal der Barbier die Geschwülste oder Apostemata in manchen Fällen aufschnitt. Alles in allem dürfte er mindestens 80 Fälle von krankhaften Veränderungen der Rachenschleimhaut behandelt haben. In 13 weiteren Fällen verzeichnete das Journal einen „bösen Mund“ oder einen Schaden im Mund – in einem Fall ergänzt durch den Hinweis auf einen „Fluss“ am Zahnfleisch. Auch die 3 Fälle von „Schorbuck“ oder „Schorbuich“ gehören in diesen Kontext. Wie schon die ergänzende Bemerkung „im Munde“ bei zweien von ihnen erkennen lässt, wurde der Scharbock oder Skorbut damals vor allem mit massiven Zahnfleischläsionen assoziiert (Mayer 2012). Rund jeder zehnte Fall in der Praxis des Barbiers entfiel somit auf krankhafte Veränderungen im Mund- und Rachenraum. Mund und Rachen waren einer lokalen Behandlung von außen mit Wässern und Salben zugänglich und fielen wohl deshalb ebenfalls in die Domäne des Chirurgen. Gleiches gilt für die 4 Fälle von Augenleiden.

Nur 5 Einträge verzeichnen Patienten mit Leiden, die damals dezidiert zu den inneren Krankheiten gezählt wurden, nämlich 4 Fälle von „colica passio“ oder „Grimmen“ des Leibs, und dazu den Fall eines Patienten mit einer Entzündung von Leib und Brust (fol. 80r). Vermutlich kamen sie in die Behandlung des Barbiers, weil bei diesen Leiden, wie in 2 Fällen ausdrücklich erwähnt, neben der Gabe von Arzneien auch die äußerliche Behandlung mit „Schmieren“ angezeigt war.

Die größte Gruppe unter den Behandelten waren die Patientinnen und Patienten mit Verletzungen, mit Wunden, Prellungen und Quetschungen, Knochenbrüchen, ausgerenkten Gliedern und dergleichen. Das Spektrum reichte vom Kind, das aufs Knie gefallen war, bis hin zu schweren Schädelverletzungen mit austretender Hirnmasse und Lähmungserscheinungen. Auf diese „traumatologischen“ Fälle entfielen insgesamt 411 Behandlungen. Dazu kommen die erwähnten achtzehn Fälle von „Schäden“, die klar auf eine Verletzung zurückgingen. Da Männer im Allgemeinen stärker durch Verletzungen gefährdet waren, dürfte dies zu dem besonders hohen Anteil der Männer in der Praxis des Barbiers beigetragen haben.

In 52 Fällen waren Knochenbrüche der Anlass für die Konsultation. Vor allem Arme und Beine waren, in den Worten des Barbiers, oft „entzwey“, aber auch gebrochene Hüften, Schultern und Rippen kamen in seine Behandlung und etliche Fälle, in denen er einen Bruch des Nasenbeins oder des Schädels vermutete. Häufig lagen Stürze zu Grunde und für etwa drei Dutzend Verletzte und Verwundete finden sich dazu genauere Angaben: 16 waren vom „Balken“ gefallen; vom „obersten Balken“ heißt es in einem Fall genauer. Gemeint war vermutlich der im Westfälischen als „Balken“ bezeichnete Heu- oder Kornboden. 2 waren vom Schornstein, 2 von einer Mauer, 7 von einem Baum und 1 aus dem Fenster gestürzt. 6 waren eine Treppe und 2 eine Leiter hinuntergefallen. Dazu kamen jene, die auf dem Eis oder auf dem Kirchhof zu Fall gekommen waren. Auch manche der 20 Kopfwunden und viele der Wunden, „Löcher“ und Quetschungen am übrigen Körper wurden vermutlich durch Stürze verursacht.

Schon die Stürze vom Heuboden und von Bäumen verweisen anschaulich auf die vielfältigen Gefahren, denen die Menschen damals in ihrer Umwelt und bei der Arbeit ausgesetzt waren. Mindestens 11 Hilfesuchende – bei anderen mag das nur nicht ausdrücklich verzeichnet sein – wurden von Pferden verletzt: Ein Pferd hatte sie in die Wange, in den Po oder in die Hand gebissen, oder mit einem Hufschlag im Gesicht, am Arm, am Bein oder im Unterleib getroffen. Einem Mädchen hatte ein Pferd den Mund „entzwei“ geschlagen und es verlor mehrere Zähne. Ein anderer Patient erlitt durch Hufschlag „Löcher“ im Kopf. Der Sohn eines Richters wurde von einem Ochsen in den Rücken gerammt, eine Magd wurde von einer Kuh ins Bein gestoßen. Über mehrere Wochen behandelte der Barbier die Wunde, die der Biss eines Schweins im Schenkel eines Patienten hinterlassen hatte.

Dazu kamen Reitunfälle, Stürze vom Pferd mit gebrochenen Knochen, Quetschungen und Wunden, vor allem an den unteren Extremitäten und am Rücken. In 4 Fällen kam es zu Verletzungen von Kopf, Schulter oder Armen, weil ein Wagen umstürzte. Eine Magd verletzte mit ihrem Wagen ein Kind schwer. Im Einzelfall fiel – vermutlich bei der Arbeit – ein schwerer Gegenstand, eine Tonne etwa oder ein Brett, auf den Kopf oder den Fuß. Ein Lehrjunge schlug sich die Finger mit einem Stockfischhammer entzwei. Vor allem kleine Kinder waren zudem durch offene Feuerstellen gefährdet. So war das Kind eines Junkers mit dem Kopf ins Feuer gefallen und die kleine Anneke Cunzwieden hatte sich das Gesicht im Feuer verbrannt.

Neben Unfällen aller Art spielte auch körperliche Gewalt eine wichtige Rolle. Wenn das Journal Löcher, Wunden, Quetschungen und dergleichen nur als solche verzeichnet, ohne den Hergang zu erläutern, können wir das im Einzelfall nur vermuten. Manche ausführlicher gehaltenen Einträge lassen jedoch wenig Zweifel. Vier Kopfwunden, die bis zum Schädelknochen reichten, dazu Schläge auf die rechte Schulter, den Arm und den Hals erlitt beispielsweise Hans Willin. Drei Wunden schlug man einem Schuhmachergesellen in den Kopf. An beiden Seiten des Kopfs erwischte es einen Knecht. Einen Finger lang war die Kopfwunde eines Manns aus Nienberge, der zudem Stiche und Schnittwunden über dem Auge, am Ohr und in der Hand erlitt. Mit zwei großen Kopfwunden, vier Stichen in den Daumen, einem zerschlagenen Ohr und gequetschten Fingern kam ein anderer Patient in Behandlung. Wenn es bei diversen weiteren Patienten heißt, dass eine Wunde „geschlagen“ oder ein Arm oder ein Auge blau „geschlagen“ worden war, lässt auch dies auf eine Fremdeinwirkung schließen. Von den insgesamt 60 Fällen von Stichwunden mögen einzelne beispielsweise auf unvorsichtiges Hantieren mit einem Messer oder mit einer Mistgabel zurückgegangen sein. In den meisten Fällen dürften die Stichwunden aber durch andere Personen zugefügt worden sein. Manche Patienten hatten sogar mehrere Stichwunden davongetragen, beispielsweise in Kopf, Rücken und Lenden.

Eine Randerscheinung waren dagegen Patienten mit Schusswunden. Nur 5 Einträge sind ihnen gewidmet, davon drei der Behandlung des zerschossenen Daumens eines gewissen Asbeck.

## Behandlung

Das Journal dokumentiert nur gelegentlich auch die Art der Behandlung. Schon die vorhandenen Beiträge eröffnen jedoch wesentliche Einblicke in das Spektrum der therapeutischen Verfahren, die ein Barbier in seiner alltäglichen Praxis anwandte und beherrschen musste, von der Amputation von Gliedmaßen und der Behandlung schwerer Kopfverletzungen bis hin zur Verschreibung und gegebenenfalls Herstellung von Salben, Wässern und anderen Arzneimitteln.

Bei Wunden stand die lokale Wundversorgung im Vordergrund. Klaffende Wunden „heftete“ er zuweilen, machte also eine Wundnaht. Bei Apostemata und anderen Geschwülsten griff der Barbier oft zum Messer, um durch einen oder mehrere Schnitte die krankhafte Materie zu entleeren, die sich unter der Haut angesammelt hatte. In diesem Sinne verzeichnet das Journal eine Reihe von Fällen, in denen er eine Geschwulst oder ein Apostem oder auch eine „böse Brust“ „eröffnete“ oder „auftat“. Vereinzelt machte er auch kleinere Amputationen. Bei einer Frau mit einem „bösen“ Daumen entfernte er ein Glied (fol. 32r), einem Mann amputierte er einen Finger (fol. 49r), und einer alten Frau im Armenhaus musste er ein Stück von ihrem rechten großen Zeh abnehmen (fol. 48v).

Wunden und chirurgisch eröffnete Geschwülste mussten nach herrschender Lehre in regelmäßigen Abständen chirurgisch versorgt werden, also, wenn nötig gereinigt und, gegebenenfalls nachdem man Salben oder dergleichen aufgetragen hatte, neu verbunden werden. Ähnliches galt für Geschwüre und nicht-verletzungsbedingte „Schäden“. Wiederholt erwähnt das Journal „Pulver“ und „Wasser“, die der Barbier bei „Schäden“ oder auch bei einer „Rose“ zur Anwendung brachte. So gab er einem Patienten mit einer stark entzündeten „Rose“ „gebrantte wasser“ und „diaphomfoligos“ (fol. 124r–v) – gemeint ist vermutlich eine Salbe, die unter der Bezeichnung „diaphonfoligos“ oder „unguentum desiccatum rubrum“ auch bei ulzeriertem Schanker zum Einsatz kam (van Hille 1706: 223). Auch krankhafte Veränderungen unter der intakten Hautoberfläche behandelte er äußerlich mit Wässern und Salben. Bei Augenleiden gab er ein „Oigenwasser“ (fol. 1r) und in manchen Fällen „schmierte“ er nach eigenen Worten, trug also offenbar eine Salbe auf (fol. 80r).

Grundsätzlich waren die Barbiere und Wundärzte im Reichsgebiet nach zeitgenössischem Verständnis für Verletzungen und Leiden zuständig, die einer äußerlichen Behandlung zugänglich waren. Die Therapie innerer Krankheiten und die innerliche Gabe von Medikamenten galt dagegen als die Domäne der studierten Ärzte und war den Barbieren vielerorts ausdrücklich untersagt. So durften die Barbiere, der Memminger Bader- und Barbiererordnung von 1566 zufolge, die ihrerseits auf der Augsburger Ordnung von 1549 gründete, innerliche Arzneimittel oder Purgantien nur mit Wissen der Stadtärzte geben (Löffler 1993: 14–16). Ähnliche Verbote finden wir in anderen Städten (z. B. Zürich, Wehrli 1927: 62). Die Mainzer Bader, Barbiere und Wundärzte mussten sich einer neuen Ordnung von 1618 zufolge gar „gäntzlichen enthalten, den Leuten einige Purgation, blutreibende [sic] vnd den Weibern schädliche Medicamenta, Clystier, oder andere treibende Artzeneyen, die zu der Cur deß Menschen, jnnerlich in den Leib gehören (wie bißhero beschehen) zu verordnen, bereyten, oder beyzubringen“ (Schweikhard 1618: 25 f.).^19^

Im Rahmen der zeitgenössischen Krankheitslehre ließ sich allerdings oft keine klare Grenze zwischen innerlichen und äußerlichen Krankheiten ziehen. Die krankhafte Materie, die sich bei Geschwülsten, Apostemata und „Flüssen“ („catarra“) unter der Haut ansammelte und bei Geschwüren nach außen abfloss, entstand auch nach Überzeugung der studierten Ärzte im Körperinneren (Stolberg 2021: 130–136). Manchmal mochte es ausreichen, der lokal angesammelten Materie durch einen oder mehrere Schnitte oder auch durch ein Zugpflaster einen Abfluss zu verschaffen. Wenn sich schon seit längerer Zeit unreine, krankhafte Materie über ein Geschwür nach außen entleerte, galt eine dauerhafte Heilung aber auch aus ärztlicher Sicht als nahezu ausgeschlossen, wenn nicht auch die eigentliche Ursache, nämlich die anhaltende Entstehung der krankhaften Materie verhindert und/oder andere Ausscheidungswege eröffnet wurden. Auch typische chirurgische Leiden, allen voran die häufigen nicht-traumatischen „Schäden“ machten vor diesem Hintergrund eine äußerliche und innerliche Therapie nötig. Nicht zuletzt erforderte die Behandlung von Wunden nach damaligem Verständnis häufig auch eine „innerliche“ Reinigung mit Purgantien, damit die Wunde nicht unreine, krankhafte Materie aus dem Körper anziehen konnte. Den Mainzer Barbieren blieb es denn auch erlaubt, „in Frantzösischen Schäden, Verwundungen, vnd andern Gebrechen zu Abheylung der Schäden, Wunden vnnd Stich, auch Heraußtreibung der Beulen vnd Geschwer notwenige Wund- vnd Lindtränck, vnd dergleichen zu ordnen und zugebrauchen“ (Schweikhard 1618: 26).

Das Journal verzeichnet wiederholt die Gabe von Mitteln zur oralen Einnahme, besonders von solchen, die der „Purgation“, also der Reinigung des Körpers von Krankheitsmaterie dienten. In manchen Fällen stand die innerliche Behandlung sogar im Vordergrund. Dem Berhardus Drosten beispielsweise gab der Barbier gegen „einen Schaden ahm Schenckel welchs die Rose Ihm gedaen hatt“ „ein purgation“, dazu „geprante wasser Ihn die 4 maß“ (fol. 41r). Dem Berhardus Oisen machte er für seine „Rose“ ein „purger drencklein“ und ein „magen puluer“; wahrscheinlich vermutete er den Ursprung der Krankheitsmaterie in einem schwachen oder verschleimten Magen (fol. 125r). Er habe ihm „gedeinet ahn allen seinen glidmasen“ mit „purgeren vnnd Schweis drenken“ vermerkte er im Fall des kranken Berent Stucker (fol. 26r). Auch anderen Patienten verabreichte er eine „Purgation“, gegen den „Schorboich“ (Scharbock) mit den charakteristischen Veränderungen an Haut und Zahnfleisch beispielsweise, die man auf den Zufluss einer scharfen Krankheitsmaterie zurückführte (fol. 69r). Einem kleinen Buben gab er „von wegen seines stolganck“ ein „drencklein“ und ein Zäpfchen (fol. 38r).

In anderen Fällen verband er oral einzunehmende Arzneien mit äußerlichen Applikationen. Die „Influmation [sic]“ des Leibs und der Brust von Junker Herman Ketteler behandelte er „miet Schmiren vnnd drencken“ (fol. 80r). Mit „drencken vnnd Olie“ heilte er einen Patienten, dem ein „Fluiß auß dem houede Ihn der borß vnnd lieb gefallen“ war (fol. 1r). Selbst die „colica passio des libes vnnd hardicheit der Seyden“, an der ein anderer Junker litt, kurierte er „miet drencken vnnd Schmiren“ (fol. 94r). Er habe der Kranken „gedeinett mit miet drencken vnnd miet Olie geschmiret dem lieb“, vermerkte er in einem anderen Eintrag (fol. 74r).

Manche Verwundete therapierte er ebenfalls innerlich und äußerlich. Einen Mann zu Nienberge, der nach einem Sturz blutunterlaufene Rippen hatte, behandelte er „miet Drenncken vnnd Schmiren“ (fol. 57v), ebenso einen Mann der sich beim Sturz von einem Balken an Kopf und Seite verletzt hatte (fol. 61v). Einem „Jungen“, der sich bei einem Sturz an Brust und Rippen verletzt hatte, „diente“ er „miett drencken, bantdoicher vnnd Olie“ (fol. 124v).

Auch der Aderlass und das Schröpfen dienten damals in erster Linie dazu, die mutmaßliche Krankheitsmaterie aus dem Körper zu entleeren. Das Journal verzeichnet nur dreimal einen Aderlass, zur Behandlung von „Schäden“ oder „Löchern“ an den Beinen oder Füßen, die zweifellos auch lokal behandelt wurden (fol. 52r, fol. 77r, fol. 111r). Schröpfen („koppen“) taucht gar nur einmal als Ergänzung zum Aderlass auf (fol. 52r). Wie schon weiter oben ausgeführt, hat der Verfasser Fälle, in denen sich seine Behandlung auf Aderlass oder Schröpfen beschränkte, aber vermutlich gar nicht verzeichnet.

## Bezahlung

Die Honorare der Münsteraner Barbiere waren nicht durch eine Gebühren- oder Taxordnung geregelt. In der Amtsrolle von 1564 hieß es nur, dass die Mitglieder der Bruderschaft den Lohn für ihre Arbeit nach Billigkeit und Gelegenheit nehmen sollten. Das im Journal verzeichnete Honorar errechnete sich offenbar entscheidend nach dem Zeitaufwand und der Schwere des Leidens oder der Verletzung. Wenn der Barbier einem Patienten oder einer Patientin über mehrere Wochen „folgte“, setzte er in vielen Fällen einen Taler pro Woche an und manchmal auch etwas mehr oder weniger. Mit „folgen“ war offenbar in der Regel ein täglicher Besuch gemeint, bei dem die Wunde oder der Schaden gegebenenfalls neu verbunden wurde. Manchmal notierte er aber auch zwei und im Fall einer schweren Mandelentzündung sogar drei Besuche an einem Tag. Gelegentlich finden sich zudem Honorarforderungen für von ihm selbst zubereitete und verabreichte Mittel.

Da der Barbier vielen Patientinnen und Patienten über mehrere Wochen „folgte“, summierten sich die durchschnittlichen Honorarforderungen zu recht beachtlichen Summen. Nur in 10 Fällen begnügte er sich mit weniger als einem Reichstaler. Dabei handelte es sich teilweise nur um die Bezahlung für Arzneien und möglicherweise sah er die betreffenden Patienten gar nicht persönlich.

Nicht alle Einträge lassen sich präzise einordnen, manchmal sind anstelle des Reichstalers (Rt) andere Münzen angeführt, ein „holländischer“ Taler beispielsweise. In Einzelfällen bezog sich das genannte Honorar zudem auf mehrere Fälle in derselben Familie. Die folgenden Zahlen geben jedoch zumindest Anhaltspunkte: Das Honorar lag in 10 Fällen zwischen 6 und 14 Schilling, in 46 Fällen bei 1 Rt, in 1 Fall bei 1,5 Rt, in 85 bei 2 Rt, in 64 bei 3 Rt, in 39 bei 4 Rt, in 5 bei 5 Rt, in 31 bei 6 Rt, in 1 Fall bei 7 Rt, in 12 bei 8 Rt, in 3 bei 10 Rt und in 3 bei 12 Rt. In rund der Hälfte der Fälle betrugen seine Honorarforderungen somit zwischen 1 und 6 Reichstalern.

In schweren Fällen, beispielsweise bei gravierenden Stichverletzungen in Bauch, Rücken oder Kopf, verdiente der Barbier noch deutlich mehr. 20 Rt bekam er für die fünfwöchige Behandlung einer offenen Kopfwunde mit Lähmungserscheinungen. 28 Rt forderte er von einem Junker, der einen offenen Schienbeinbruch erlitten hatte, 21 Rt von einem Patienten, der sich beim Sturz von der Treppe Stirn und Nase verletzt hatte und dem dabei eine Arterie geborsten war. Auch die Behandlung von Schäden und „Flüssen“ schlug im Einzelfall mit 18, 21 oder gar 26 Rt zu Buche.

Die Zahlen zeigen, dass wir keineswegs davon ausgehen dürfen, dass die Behandlung durch einen Barbier grundsätzlich deutlich billiger war als die eines studierten Arztes. Die im Journal verzeichneten Honorare lagen vielmehr ziemlich genau in der Größenordnung, die der Münsteraner Rat für die studierten Ärzte für angemessen hielt. Da „mancher scheugetragen, die Doctores Medicinae zu consultiren propter incertam taxam“, beschloss der Rat 1610, dass die Ärzte für einen Besuch 4 Schillinge beziehungsweise für eine einwöchige Behandlung 1 Reichstaler erhalten sollten.^20^ Hiob Finzel forderte im ausgehenden 16. Jahrhundert als Stadtarzt von Zwickau von den meisten Kranken sogar nur wenige Groschen (Stolberg 2019).

Manchmal wurde der Barbier teilweise oder ganz mit Naturalien entlohnt, mit Wein oder Getreide vor allem, im Einzelfall freilich auch mit zwei Scheffeln Äpfel oder mit Stoff für ein Paar Hosen.

Präzise Aussagen über das jährliche Gesamteinkommen lässt das Journal aufgrund der lückenhaften Angaben nicht zu. Aus den Einträgen, die Zahlen benennen, lässt sich aber schließen, dass sich die Honorarforderungen auf jährlich mindestens rund 100 Reichstaler beliefen. Das war eine stattliche Summe, zu der man vermutlich noch zahlreiche kleinere Beträge für Aderlässe, Zahnziehen und einmalige, sofort bezahlte Konsultationen hinzurechnen muss. Die genauere Untersuchung des Journals macht allerdings eine sprachliche Besonderheit deutlich, die einerseits die Berechnung des tatsächlichen Einkommens zusätzlich erschwert, andererseits aber auch die Funktion des Journals als solche erhellt. In zahllosen Einträgen verzeichnete der Barbier nämlich einen Geldbetrag, den er „verdient“ hatte, oder schrieb nur kurz „davon“. Das heißt jedoch nicht, dass er dieses Geld tatsächlich bekam. Offenbar durfte er nämlich nicht erwarten, dass man seine Forderungen stets anstandslos beglich. Mit „verdient“ bezeichnete er die Summen, die er forderte und die ihm nach seiner Überzeugung zustanden, so wie wir heute noch beispielsweise von einem Lob oder einer Strafe sprechen, die jemand für sein Tun „verdient“ hat. Der gleiche Begriffsgebrauch zeigt sich auch in den Ratsprotokollen, denen zufolge beispielsweise der Barbier Hermann Hölscher senior einmal drei Reichstaler für die Behandlung eines Verwundeten forderte und meinte, er habe „mehr dan 2. mahl sovil dran verdienet.“^21^

Immer wieder finden sich neben dem „verdienten“ Honorar ergänzende Angaben zu den Geldern oder Getreidemengen, die der Barbier tatsächlich „empfangen“ hatte, nicht selten erkennbar später in anderer Tinte ergänzt. Andere Einträge benennen Teilbeträge, die bereits entrichtet wurden, und weitere, die für einen zukünftigen Termin versprochen waren oder die man noch schuldig war (und möglicherweise für immer schuldig blieb). Zuweilen heißt es auch ausdrücklich, man habe sich „verglichen“, sich also auf einen niedrigeren Betrag geeinigt. Vermutlich spiegeln solche Einträge Diskussionen und Auseinandersetzungen über das angemessene Honorar wider, wie sie ohne eine verbindliche Gebührenordnung kaum zu vermeiden waren. Die Amtsrolle traf entsprechende Vorkehrungen. Wenn sich die Parteien nicht einig wurden, sollten die Zunftverweser das Honorar festlegen. Im Einzelfall trat auch der Rat selbst als Schlichter auf, wenn ein Barbier auf einem höheren Honorar beharrte. Er befragte den Ratsprotokollen zufolge zuweilen sogar Nachbarn und Bedienstete als Zeugen beispielsweise über die Schwere der betreffenden Verletzung.^22^

## Scheren

Der Name „Barbier“ leitet sich bekanntlich vom lateinischen „barba“ für Bart ab und das Scheren von Bart und Haaren war auch im 17. Jahrhundert noch eine charakteristische Tätigkeit der Barbiere. Einige Einträge am Ende des Journals machen deutlich, dass sich auch dessen Verfasser mit dem Scheren abgab. Er notierte zu einer Reihe von namentlich genannten Kunden Vereinbarungen über einen jährlichen „Scherlohn“. Es ist nicht ersichtlich, wie häufig er diese Männer schor und inwieweit er auch das Kopfhaar schnitt, aber andere zeitgenössische Quellen lassen erkennen, dass es üblich war, den Bart einmal oder allenfalls zweimal in der Woche zu rasieren (Grosser 2022: 325). Die Einträge beginnen, offenbar im Rückblick geschrieben, mit Ostern 1601. Damals bekam er von dem Vikar der Kirche in Überwasser einen halben Taler „vor Scherlonn“ (117v). Vermutlich noch im gleichen Jahr begann er Meister Berent Schauvedink zu scheren, von dem er für zwei Jahre einen Ortstaler erhielt (fol. 121v). Für 1602 sind drei weitere solche Vereinbarungen vermerkt. Der Sekretär des Domkapitels Johann Tegelter und ein Lizentiat zahlten ihm je einen Reichstaler als „Scherlon“. Von einem gewissen Johan Jodefeld junior bekam er an Pfingsten einen Scheffel Roggen oder Weizen als Scherlohn. Das entsprach, wie zwei Einträge im Journal zeigen, ungefähr einem halben Reichstaler (fol. 116v). 1604 begann er gegen einen Reichstaler den Doktor Tegelter^23^ und einen weiteren „Herren“ für einen Scheffel Roggen und ein paar Hühner jährlich zu scheren (fol. 117r). Im November 1607 nahm er den Drost von Sassenberg, Oberst Alexander von Velen, als Kunden zum Scheren an, der ihm alle Jahre einen Malter Roggen zahlte (fol. 117v). 1610 begann er Heinrich Asbeck und 1611 den Dekan Caspar Doerhoiff gegen eine jährliche Zahlung von einem halben bzw. einem ganzen Reichstaler zu scheren (fol. 118r). Im Oktober 1615 vereinbarte er, Johann Boichorß gegen zwei Scheffel Weizen jährlich zu scheren (fol. 118v). Einen nicht näher bezifferten, noch ausstehenden Scherlohn vermerkte er gesondert zu seiner Honorarforderung über 21 Rt an Drost Ketteler in Dülmen (fol. 120r). Wie im Fall von Aderlass und Zähnereißen ist zu vermuten, dass er „Laufkundschaft“, die nur gelegentlich seine Dienste forderte, gar nicht erst im Journal verzeichnete.

## Schluss

Das hier vorgestellte und untersuchte Journal eröffnet vielfältige und reichhaltige Aufschlüsse über die Praxis eines handwerklich gebildeten Wundarztes im frühen 17. Jahrhundert. Vieles spricht dafür, und dies macht die Quelle umso wertvoller, dass der Verfasser, soweit Verallgemeinerungen überhaupt möglich sind, ein typischer Vertreter seiner Berufsgruppe war. Er betätigte sich, wie die Barbiere das üblicherweise taten, auch als Bartscherer, führte keine größeren Operationen durch und trat nicht durch gedruckte Veröffentlichungen hervor.^24^ Allenfalls lässt die Tatsache, dass er auch zur Behandlung schwerer Verletzungen herangezogen wurde und selbst Kranke und Verletzte aus den höchsten Kreisen seine Hilfe suchten, vermuten, dass er in Münster eher zu den führenden Vertretern seiner Zunft zählte.

Verallgemeinernde Schlussfolgerungen lassen sich mangels vergleichbarer ähnlicher Quellen nur mit der gebotenen Vorsicht ziehen. Vier wesentliche Ergebnisse dieser Untersuchung seien jedoch abschließend und zusammenfassend genannt:

1. Wenn die Begriffe „Barbier“, „Wundarzt“ und „Chirurg“ für Vertreter dieser handwerklich ausgebildeten Gruppe in der historischen Forschung weitgehend synonym gebraucht werden, entspricht das dem zeitgenössischen Begriffsgebrauch. Es drohen jedoch gravierende Missverständnisse, wenn wir unser heutiges Verständnis von „Chirurgie“ auf die Frühe Neuzeit übertragen. Der damalige Begriffsgebrauch stand den etymologischen Wurzeln deutlich näher als heute: „Chirurgie“ leitet sich bekanntlich von den griechischen Wörtern für „Hand“ und „Arbeit“ oder „Werk“ ab. „Chirurgie“ war so gesehen zunächst einmal nur ein Bereich der Medizin, der den Einsatz der Hände erforderte. Die wenigen großen, invasiven Eingriffe, die damals möglich waren – im Wesentlichen die Operation von Blasensteinen und Hernien – blieben weitgehend einer kleinen Gruppe von Spezialisten vorbehalten. Sie spielten in der alltäglichen wundärztlichen Praxis keine Rolle. Selbst die Behandlung von Wunden und Knochenbrüchen machte nur einen Teil der im Journal dokumentierten Praxis aus. Viele, ja wahrscheinlich die meisten Ratsuchenden kamen wegen Geschwüren, Geschwülsten, Abszessen und ähnlichen krankhaften Veränderungen auf, in und unter der „wunden“ Haut oder den Schleimhäuten.
2. Das Journal zeigt, dass die Klagen damaliger Ärzte und Apotheker, dass die Barbiere auch innerlich, mit oral einzunehmenden Arzneien behandelten, nicht ganz unberechtigt waren. Es dokumentiert in diversen Fällen die Gabe von Mitteln zur oralen Einnahme. Teilweise stellte der Barbier diese selbst her und verlangte dafür ein Entgelt. Damit überschritt er aber nicht notwendig die Grenzen seiner beruflichen Domäne. Eine „innerliche“ Behandlung war auch bei vielen „äußerlichen“ Krankheiten angezeigt. Sie galt in manchen Fällen sogar als unverzichtbar und wurde als solche von den Obrigkeiten akzeptiert: Geschwülste und Geschwüre entstanden nach verbreiteter Überzeugung durch den Zufluss von Krankheitsmaterie aus dem Körperinneren. Die Natur bediente sich ihrer, um den Körper von der Krankheitsmaterie zu befreien. Der Wundarzt konnte diesen Vorgang unterstützen, indem er Geschwülste aufschnitt und Geschwüre gezielt an der Abheilung hinderte, solange noch unreine Materie abfloss. In vielen Fällen war es aber auch angezeigt, der Krankheitsmaterie beispielsweise durch Abführ- oder Brechmittel einen alternativen Ausweg zu eröffnen. Denn eine dauerhafte Heilung war letztlich nur zu erwarten, wenn es gelang, die weitere Entstehung der Krankheitsmaterie zu verhindern, und das erforderte oft zwangsläufig die Gabe von Mitteln, die im Körperinneren wirkten, die beispielsweise einen schwachen, verschleimten Magen stärken und reinigen konnten, der ständig neue Krankheitsmaterie produzierte.
3. Die zahlreichen Erläuterungen zu den Honorarforderungen verweisen auf einen in der historischen Forschung bislang wenig beachteten, aber für die Qualität der therapeutischen Beziehung folgenreichen Unterschied zwischen der ärztlichen und der wundärztlichen Praxis: Manche Kranke vor allem in den oberen Schichten, holten den studierten Arzt bei inneren Leiden immer wieder zu sich, damit er sie im Krankheitsverlauf weiter betreute und seine Behandlung dem jeweiligen Zustand anpasste. Wie das Praxisjournal des Zwickauer Stadtarztes Hiob Finzel aus dem späten 16. Jahrhundert zeigt, begnügten sich viele Ratsuchende jedoch mit einer einmaligen Konsultation. Der Arzt stellte seine Diagnose und gab oder verschrieb ihnen Arzneien. Im Idealfall nahmen sie die Arzneien ein und wurden nach einer gewissen Zeit wieder gesund. Besserte sich ihr Zustand nicht, so lag die Vermutung nahe, dass die Diagnose nicht richtig oder zumindest die verordnete Behandlung nicht ausreichend wirksam war. Dann konnte man mit demselben Arzt einen weiteren Versuch wagen. Man konnte sein Glück aber auch bei einem anderen Arzt versuchen. Und genau das taten viele Kranke den Klagen der Ärzte zufolge (Stolberg 2019). Ganz anders, das führt das hier untersuchte Journal eindrücklich vor Augen, gestaltete sich die chirurgische Praxis. Der Barbier „folgte“ vielen Patientinnen und Patienten über Wochen, besuchte sie regelmäßig. Das lag in der Natur der chirurgischen Behandlung. Verletzungen, Geschwüre und mit dem Messer eröffnete Geschwülste erforderten oft regelmäßige Verbandswechsel, das erneute Auftragen von Salben und anderen äußerlich anwendbaren Mitteln. Zugleich waren damit im Vergleich zu den oft nur gelegentlichen oder einmaligen Besuchen bei einem „Doctor“ ganz andere Voraussetzungen für die Entstehung einer längeren und entsprechend intensiveren therapeutischen Beziehung gegeben.
4. Der Barbier behandelte Hilfesuchende aus allen Gesellschaftsschichten. Auch die Oberschichten, die damals bei inneren Krankheiten zunehmend einem studierten Arzt den Vorzug gaben, vertrauten in chirurgischen Fällen einem „nur“ handwerklich ausgebildeten Praktiker, der ausweislich seiner Orthographie und der unkonventionellen Schreibweise der wenigen lateinischen Fachbegriffe, die er gebrauchte, offenbar nicht besonders gebildet und somit wohl kaum mit dem gelehrten lateinischen Schrifttum der Zeit vertraut war. Die vielen Fälle, in denen sich das Honorar auf 3, 4, oder 5 Taler oder noch mehr belief, machen gleichzeitig deutlich, dass die Behandlung durch einen Barbier nicht unbedingt weniger kostete als die eines studierten Arztes. Oft war sie wegen des größeren Zeitaufwands für die regelmäßigen Visiten sogar deutlich kostspieliger. Die These, die Barbiere seien gewissermaßen die Ärzte des „gemeinen Manns“ gewesen, lässt sich auch im Blick auf die Honorare nicht aufrechterhalten. Offensichtlich entschieden sich die Zeitgenossen nicht wegen der voraussichtlich geringeren Honorarforderungen dafür, lieber einen handwerklich gebildeten Wundarzt als einen gelehrten Arzt zu konsultieren. Entscheidend waren die Kenntnisse und ganz buchstäblich handwerklichen Fähigkeiten, die für die Behandlung von Verletzungen und anderen äußerlich zu behandelnden Leiden erforderlich waren und über die die gelehrten Ärzte in Deutschland, anders als in Italien, damals in der Regel (noch) nicht verfügten.

## Footnotes

1 Von Brunn (1921) gibt im Quellenanhang unter anderem einschlägige „Rollen“ aus Stralsund und Rostock wieder. Sie wurden auch als „Ruhlen“ bezeichnet (Stadtarchiv Münster (StAM), Ratsprotokoll (RP) 1602, S. 46); vgl. dazu das englische „rule“ für „Regel“.

2 Siehe Groß (1911: 109–127); von Brunn (1921); Mewald (1962); Brandenburger & Nissen (1964, 23–38); Nauck (1965); Pfeiffer (1966); Eckardt (1991); Fischer (1991); Münkle (1992); Löffler (1993); Helber (1995: 75–147); Besl (1996); Dieminger (1999); Plank (1999); Bühler (1999); Maisch (2011); Beutter (2011); Kinzelbach (1994); Kinzelbach (2014); Kinzelbach (2017); Kinzelbach (2022); Dross (2022); Grosser (2022).

3 Die einzige nennenswerte Ausnahme ist für das deutsche Reichsgebiet Robert Jüttes Untersuchung der Praxis des Wundarztes Gerhard Eichhorn in Köln (Jütte 1989; Jütte 1991: 102–106). Allerdings bezieht sich seine Analyse nur auf jene insgesamt 60 Fälle, über die Eichhorn zwischen 1625 und 1638 den Zunftmeistern berichtete und die deshalb im sogenannten „Beleidbuch“ der Kölner Barbiere Aufnahme fanden. Die Zunftmeister mussten aber nur dann eingeschaltet werden, wenn Lebensgefahr, eine Lähmung oder eine Amputation drohte, oder wenn eine Wunde sich nach viermaligem Verbinden nicht gebessert hatte. Wenn Eichhorns Angabe, er sei im Jahr 1634/35 von 200 Patienten konsultiert worden, stimmt und das Jahr 1634/35 einigermaßen repräsentativ für seine Praxis war, hätten die 60 Fälle nur rund 2,5 % der in diesem Zeitraum insgesamt von ihm behandelten Fälle ausgemacht, und zwar in erster Linie die schweren verletzungsbedingten Fälle, die für seine Praxis insgesamt nicht repräsentativ waren.

4 Linda McCray Beier (1992) hat das *caseboo*k des *barber-surgeon* Joseph Binns in London mit 670 Eintragungen in den Jahren zwischen 1633 und 1663 untersucht. Die englischen *barber-surg*eons unterschieden sich jedoch deutlich von den deutschen Barbieren, und Binns war, McCray Beier zufolge, zudem alles andere als ein typischer, durchschnittlicher Vertreter seiner Zunft.

5 StAM A XI, Nr. 249, foll. 49r–56v; weitere Abschriften im gleichen Band und in Nr. 250 und Nr. 251.

6 StAM, RP 1608, fol. 290v.

7 Landesarchiv Nordrhein-Westfalen, V 502/Verein für Geschichte und Altertumskunde Westfalens, Abteilung Münster (Dep.), Manuskripte, Nr. 293. Auf den umfangreichen chronologisch geordneten Hauptteil folgen gegen Ende noch einzelne Einträge zu Fällen und Kunden aus unterschiedlichen Jahren. Soweit aus der Struktur der mit Fäden zusammengebundenen Faszikel erkennbar, ist der Band bis auf das erste (herausgeschnittene) Blatt vollständig überliefert. Ganz am Schluss finden sich, heute mit foll. 123–126 paginiert, vier aus zwei in der Mitte gefalteten Bögen gebildete Blätter mit Eintragungen aus dem Jahr 1611. Vermutlich waren diese Blätter ursprünglich in der Mitte des Faszikels mit den foll. 109–122 platziert, lösten sich heraus und wurden dann falsch eingebunden.

8 StAM, RP 1618, foll. 117v–118r.

9 Sicher ausschließen lassen sich nur Bernd Westeken, der 1602 erst um die 22 Jahre alt war (er ist in StAM, Acta judicialia, Nr. 746, 1622 als ca. 44-jähriger Zeuge dokumentiert), und einen der beiden erwähnten Barbiere mit Namen Bernd Lange, der 1609 verstarb (StAM, RP 1609, fol. 99v).

10 StAM, A XI Nr. 25, fol. 67r, 04.03.1602.

11 Manche Einträge sind nicht eindeutig individualisiert, wenn etwa ohne Nennung des Vornamens nur der „Sohn“ oder die „Tochter“ oder gar der Knecht eines namentlich genannten Mitbürgers angegeben ist.

12 Die Zahl der Kinder dürfte noch höher gewesen sein, denn zahlreiche Einträge verweisen auf die „Tochter“ oder den „Sohn“ eines namentlich genannten Mitbürgers. Größere Kinder könnten sich darunter ebenso finden unverheiratete Frauen und Männer, die noch im Haus der Eltern lebten.

13 Es gibt im Einzelfall Unsicherheiten. Wenn ein Vorname von einem „von“ gefolgt ist, ist nicht immer klar, ob das Wort nach dem „von“ ein Eigenname oder ein Ortsname ist.

14 Universitätsarchiv Tübingen, UAT 20/9,1 Nr. 3, Testimonium vom 05.08.1608; zur Behandlung der Franzosenkrankheit im Allgemeinen s. a. Stein (2003) und Stein (2021).

15 Einschließlich eines Gehilfen oder Lehrlings („Jungen“), von dem der Chirurg nur meinte, er habe ihn am „Gemecht“ geheilt.

16 Beispielsweise fol. 8r, fol. 39r, fol. 90v und fol. 94v; zum weiblichen Schamgefühl siehe auch Jütte (1992).

17 Landesarchiv NRW Abteilung Westfalen, B 151 Studienfonds Münster, Akten, Nr. 9636.

18 Wellcome Collection, London, Western Manuscripts 93, fol. 42r.

19 Ähnliche Verordnungen ergingen 1510 in Nördlingen (Kinzelbach 2017: 275) und seit 1532 wiederholt in Augsburg (Stein 2003: 159).

20 StAM RP 1610, fol. 230v, „Salarium der medicinae doctorum“; den Patienten wurde allerdings geraten, sich im Vorfeld mit dem Arzt darüber zu verständigen, dass er sich auch tatsächlich mit diesem Honorar begnügen würde.

21 StAM RP 1611, fol. 204r.

22 StAM, RP 1609, fol. 275r–v; StAM, RP 1611, fol. 249r.

23 Es könnte sich auch um eine versehentliche doppelte Eintragung zum erwähnten Sekretär des Domkapitels handeln.

24 Keiner der namentlich bekannten Münsteraner Chirurgen jener Zeit ist als Autor von chirurgischen Schriften belegt.
